# Digital interventions for depressive symptoms: a randomized clinical trial

**DOI:** 10.47626/2237-6089-2024-1006

**Published:** 2025-11-05

**Authors:** Júlio César Bebber, Bruno Braga Montezano, Analise de Souza Vivan, Thyago Antonelli-Salgado, Kyara Rodrigues de Aguiar, Aline Zimerman, Augusto Ossamu Shintani, Marta Braga Ryff Moreira, Roberta Campos, Lidiane Rodrigues, Guilenne Frisina Zaffari, Glória Mallmann, Rafaela Fernandes Pulice, Victória Chiodelli Senger, Juliana Rosendo Vargas, Camila Zimmer, Mirian Cristina dos Santos Amaral, Gabriel Gonçalves Veloso, Giancarlo Franceschi Dalla Vecchia, Júlio César Bisognin Lopez, André Russowsky Brunoni, Francisco Diego Rabelo-da-Ponte, Ives Cavalcante Passos, Daniela Tusi Braga

**Affiliations:** 1 Hospital de Clínicas de Porto Alegre Centro de Pesquisa Clínica Porto Alegre RS Brazil Laboratório de Psiquiatria Molecular, Centro de Pesquisa Experimental (CPE), Centro de Pesquisa Clínica (CPC), Hospital de Clínicas de Porto Alegre (HCPA), Porto Alegre, RS, Brazil.; 2 Instituto Nacional de Ciência e Tecnologia Translacional em Medicina Porto Alegre RS Brazil Instituto Nacional de Ciência e Tecnologia Translacional em Medicina (INCT-TM), Porto Alegre, RS, Brazil.; 3 Universidade Federal do Rio Grande do Sul Faculdade de Medicina Departamento de Psiquiatria Porto Alegre RS Brazil Programa de Pós-Graduação em Psiquiatria e Ciências do Comportamento, Departamento de Psiquiatria, Faculdade de Medicina, Universidade Federal do Rio Grande do Sul (UFRGS), Porto Alegre, RS, Brazil.; 4 Universidade Luterana do Brasil Faculdade de Medicina Canoas RS Brazil Faculdade de Medicina, Universidade Luterana do Brasil (ULBRA), Canoas, RS, Brazil.; 5 Universidade Federal do Pampa Faculdade de Medicina Uruguaiana RS Brazil Faculdade de Medicina, Uruguaiana, Universidade Federal do Pampa (UNIPAMPA), Uruguaiana, RS, Brazil.; 6 Universidade de São Paulo Departamento de Psiquiatria da Faculdade de Medicina São Paulo SP Brazil Departamento de Psiquiatria da Faculdade de Medicina, Universidade de São Paulo, São Paulo, SP, Brazil.; 7 King's College London Institute of Psychiatry Social, Genetic & Developmental Psychiatry Centre United Kingdom Social, Genetic & Developmental Psychiatry Centre, Institute of Psychiatry, Psychology & Neuroscience, King's College London, United Kingdom.

**Keywords:** Depression, depressive symptoms, digital intervention, group cognitive-behavioral therapy, smartphone applications

## Abstract

**Objective:**

Depression is a prevalent mental health condition with a significant global burden, yet treatment coverage remains limited. Digital interventions offer a promising avenue for expanding access to evidence-based interventions.

**Methods:**

In a three-arm randomized clinical trial, we evaluated the efficacy and safety of an app-based intervention and an online group cognitive behavioral therapy (GCBT) to reduce depressive symptoms compared to a waiting list control (WLC). Participants (N = 109) with PHQ-9 scores ≥ 9 were randomized into three groups. Informed consent was obtained. The primary outcome, depressive symptoms, was assessed at baseline and every 4 weeks over 12 weeks. Secondary outcomes included anxiety symptoms, loneliness perception, and treatment-related adverse effects. We used one-tailed Student's t-tests and Mann-Whitney U tests, adjusting p-values for false discovery rate. Statistical significance was set at 5%. ClinicalTrials.gov identifier: NCT05450614.

**Results:**

After excluding dropouts, 58 participants remained (28 app; 19 GCBT; 11 WLC). Most were women (app: 86%; GCBT: 89%; WLC: 100%) and identified as white (app: 61%; GCBT: 63%; WLC: 82%), aged 36 to 39, with high income and education. Only GCBT showed a significant reduction in anxiety (*t*(23.92) = 2.20, p = 0.019; p_adj_ = 0.038; Cohen's *d* = 0.81, 95%CI [0.17, ∞). The remaining comparisons were not statistically significant.

**Conclusion:**

While only GCBT showed significant improvement in anxiety symptoms, both treatments showed trends toward depressive symptom reduction. High dropout rates and a small sample may have impacted results. Further research should assess the long-term impact and scalability of digital interventions in mental health.

## Introduction

Major depressive disorder (MDD) is a highly prevalent mental disorder with a lifetime prevalence of 7.5% for males and 13.6% for females.^1^ Since 1990, there has been no observed reduction in its prevalence or impact on the global burden of diseases.^2^ MDD is one of the leading causes of disability worldwide.^3^ The COVID-19 pandemic has further exacerbated this situation, with a 27.6% increase in the prevalence of MDD over the first year.^4^

However, there is limited treatment coverage. According to a systematic review, which includes data from 84 countries between 2000-2019, the MDD treatment coverage in health services ranged from 51% [95% UI 20%, 82%] in high-income locations to 20% [95% UI 1%, 53%] in low- and lower-middle-income locations.^5^ Barriers to adequate assistance include a lack of investment in mental health policies, healthcare professional shortages, and social stigma.^6^ This treatment gap has broad consequences, affecting well-being and imposing economic and social burdens.^7^ It also exacerbates comorbidities, including heart disease,^8^ anxiety,^9^ loneliness,^10,11^ and cancer.^12^

Smartphones have become a potent tool for extending the reach of traditional treatment, due to their portability, wireless capabilities, affordability, and instant internet access/connectivity from anywhere. Globally, the number of smartphones in use has reached approximately 6.94 billion, showcasing a widespread adoption of this technology. Notably, penetration rates are significant, standing at 81.6% in the United States and 66.6% in Brazil.^13^ These statistics reflect a continual year-over-year increase in smartphone users worldwide. Digital psychiatry has emerged, initially through the internet and computers, and later through the use of smartphones. While there are numerous advantages to delivering psychotherapy via computers, obstacles related to accessibility have been encountered,^14^ which can be partly resolved through the benefits offered by smartphones and their applications (apps).

Although numerous mental health-focused apps are available for download in virtual stores, most of them lack theoretical foundations and evidence to support their use in clinical practice. A systematic review examined 293 mobile apps for anxiety and depression, finding that only 10 (6.2%) had published data supporting their effectiveness.^15^ Despite this, the field is growing. Recently, the app Rejoyin was approved by the FDA as an add-on prescription for managing depressive symptoms.^16^ Furthermore, a recent systematic review and meta-analysis demonstrated the effectiveness of smartphone apps in alleviating depressive symptoms. The authors selected 13 randomized clinical trials, covering 16 intervention apps and a total of 1470 participants. Their findings showed that mobile app interventions were significantly associated with reductions in depressive symptoms, with a medium effect size (SMD 0.50; 95%CI 0.40 to 0.61).^17^

The app under study equips users with critical strategies based on cognitive-behavioral therapy (CBT) to reduce depressive symptoms. This RCT aimed to evaluate the app's efficacy and safety in reducing depressive symptoms over 12 weeks (primary outcome). Additionally, an online group cognitive-behavioral therapy (GCBT) was included for comparison. Secondary outcomes included a reduction in anxiety and loneliness symptoms, as well as any adverse effects of the proposed treatments.

## Methods

### Design

**Figure 1:**
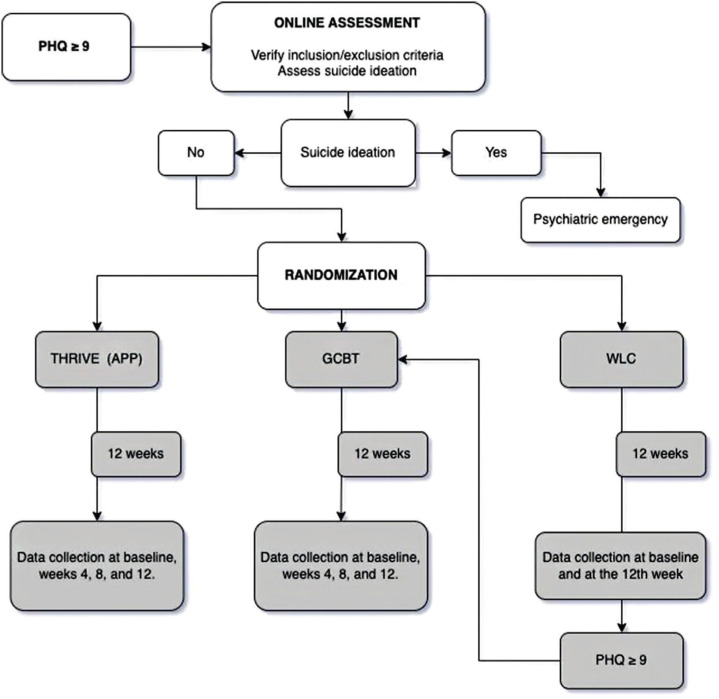
Trial protocol. GCBT = group cognitive-behavioral therapy; THRIVE (APP) = app-based intervention; WLC = waiting-list control.

This is a three-arm randomized clinical trial. We randomly assigned participants to one of the study groups to compare the effectiveness of two online interventions, app-based intervention and group cognitive-behavioral (GCBT), against a waiting list control (WLC). Figure 1 provides the trial protocol. The study received approval from the ethics committee at Hospital de Clínicas de Porto Alegre and informed consent was obtained from all participants included in the study. The report is in accordance with the CONSORT 2010 Statement.^18^ The original study protocol, as registered in ClinicalTrials.gov (NCT05450614), described the trial as a non-inferiority study. However, this approach was not adopted in the final analysis due to the high sample size requirement for achieving statistical significance in non-inferiority trials. Given the limitations in recruitment, the study design was adjusted accordingly.

### Participants

We invited participants with Patient Health Questionnaire (PHQ-9) scores of 9 or higher from a previous online survey conducted by our research group.^10^ They were contacted via email, WhatsApp, and phone calls. Furthermore, we promoted the study through our research team's social media platforms and mental health institute networking platforms.

Eligibility criteria included owning a compatible smartphone, being between 18-65 years old, having a score ≥ 9 on PHQ-9 at baseline, residing in Brazil, and having proficiency in Portuguese. Individuals were ineligible for participation if they were pregnant, had visual impairments that made it impossible to use the app, were diagnosed with bipolar disorder, schizophrenia, schizoaffective disorder, intellectual disability, had a history of alcohol or drug abuse within the past year, or had a suicide risk <6 (Mini-International Neuropsychiatric Interview [MINI]). Additional coexisting medical conditions were considered, except when they were degenerative (such as dementia or multiple sclerosis) in a manner that could impact interaction with the application. The utilization of psychotropic medications and external psychotherapy was allowed throughout the study. The participants were evaluated by experienced mental health professionals, and diagnostic assessments were conducted using the MINI.^19^

### Procedure

This was an entirely online study. Participants who responded to the invitation or were recruited through social media promotion were invited to an initial assessment via video or phone call with a member of our research team. During this assessment, the study was explained in detail, and the inclusion and exclusion criteria were verified.

Individuals who remained potentially eligible waited until the minimum number of participants for stratified randomization. The random allocation sequence was generated by scripts written in the R programming language. Stratified randomization was employed to ensure a balance between groups concerning depression severity level in the PHQ-9. The researchers conducted this assignment process using the predetermined allocation ratio. Stratified randomization ensured that participants were evenly distributed across intervention groups within each stratum. Stratification for this study relied on PHQ-9 scale scores, categorizing participants into three predetermined severity groups, consistent with established literature: 10-14 indicating moderate symptoms, 15-19 indicating moderate to severe symptoms, and 20-27 indicating severe symptoms.^20^

The study involves four randomizations at different points in time, with two randomizing GCBT and app-based intervention group, and the other two WLC and app-based intervention groups. The screening phase coincided with the intervention phase in the study, allowing researchers to assess participant eligibility while implementing interventions concurrently. This approach optimizes time and resources. The study extensions lasted for approximately 18 months. The screening phase began in July 2022 and lasted for a year. The intervention phase, which encompassed GCBT, app-based intervention, and Waiting List Control (WLC), started at different times, as mentioned earlier, beginning in September 2022 and ending in December 2023.

### Intervention conditions

#### App

Thrive: digital mental health is a mobile app available on iOS and Android, developed by the Institute of Neurosciences and Cognitive Therapies (INTC), whose partners are also co-authors of the study. Designed to integrate Cognitive Behavioral Therapy (CBT) principles and techniques. Thrive provides participants with effective tools and strategies for self-improvement, particularly in managing depressive symptoms. The app consists of five key dimensions: psychoeducation, providing information on mental health and well-being; symptom monitoring, allowing users to regularly track their emotional and physical state; behavioral activation, encouraging engagement in enjoyable and meaningful activities; thought recording, to identify and restructure dysfunctional thought patterns; and general tools, such as a gratitude diary, coping strategies cards, muscle relaxation exercises, and diaphragmatic breathing techniques.

Developers designed Thrive to be customizable and user-friendly, allowing individuals to personalize features like symptom-tracking reminders or entries in the gratitude diary. With these evidence-based techniques and the input of mental health professionals, the app aims to offer a practical and scientifically grounded approach to mental health management. Screens from the app, which illustrate these features, are shown in Supplementary Figures S1 and S2.

The intervention was conducted over 12 weeks. Participants in the app group were given access to the app and instructions on how to use it. In addition to the user guide within the app interface, participants received a weekly guided task via WhatsApp messages, including text and video content. These tasks were aligned with the principles of CBT for depression. Moreover, users had access to a personal curator, who, despite being an experienced psychologist, assisted participants in the Thrive app group solely by resolving questions related to the use of the app. This ensured that support was focused on facilitating user interaction with the app, rather than providing direct psychological counseling.

#### Group cognitive-behavioral therapy (GCBT)

Participants in the GCBT group attended weekly 90-minute online sessions organized into three groups of up to 10 members. These groups were staggered in their start times due to logistical constraints and each session was facilitated by a therapist and a co-therapist. It is noteworthy that the therapists were psychologists with expertise in cognitive-behavioral therapy. The therapist played an active role in facilitating group dynamics, leading sessions according to the protocol, ensuring that all participants’ needs were addressed, and managing session time allocation. Meanwhile, the co-therapist observed and tracked each member's participation and engagement. In cases where a participant experienced heightened distress during a session, they could receive individualized attention from the co-therapist, enabling the group to continue smoothly without interruptions.

In the literature, there is no single protocol for the treatment of depression through GCBT. Therefore, a systematic protocol was developed for this study to structure and standardize this intervention.^21^ The group intervention consisted of 12 sessions, following the same CBT techniques offered by the app-based intervention. The only difference in interventions was that GCBT was conducted by a professional synchronously. The protocol included a variety of content, such as information about the manifestations of depression and its symptoms, aiming to assist patients in recognizing and monitoring their own emotional, behavioral, and cognitive changes. Biological aspects of depression, such as genetic, environmental, and neurobiological factors, were addressed. Additionally, the cognitive-behavioral model of depression was explored, including typical cognitive distortions that occur in depressed patients. Subsequent sessions covered behavioral activation therapy, self-monitoring of emotions, behaviors, and dysfunctional thoughts, and the use of cognitive techniques to correct distortions, aiming to reduce or eliminate depressive symptoms. Topics such as the importance of healthy habits, the influence of depression on family, and strategies for relapse prevention were also discussed. Each session included practical exercises corresponding to the topics covered, both for completion during the session and as homework assignments, aiming to consolidate learning and improve symptoms.

#### Waiting list control (WLC)

The WLC allowed participants to continue their treatment as usual during 12 weeks. At the end of the period, a reassessment of symptoms was conducted using the PHQ-9 scale. Participants who continued to exhibit depressive symptoms (PHQ ≥ 9) were referred to a GCBT protocol, even though they had completed their participation in the study.

### Outcome assessments

Primary outcome (PHQ-9) was measured at baseline and every 4 weeks up to 12 weeks. We assessed secondary outcomes at the same time points, including anxiety symptoms (Generalized Anxiety Disorder 7-item [GAD-7]), perception of loneliness (University of California, Los Angeles - Loneliness Scale [UCLA-3 item]), app usage metrics, and adherence parameters (such as app usage and the number of GCBT sessions attended).

The Negative Effects Questionnaire (NEQ) was administered at each assessment point following the baseline, enabling the evaluation of treatment-related side effects. The NEQ is proposed as a useful instrument for investigating potential side effects in psychological treatments.^22^ The item descriptions are available at Supplementary Table S1. Throughout the study, suicide risk was assessed using the final question of the PHQ-9 scale; if there was a positive response, the patient was contacted for further evaluation. Suicidality following clinical assessment leading to the exclusion of participants and referral to an emergency mental health service.

### Adherence

In the app-based intervention group, adherence was defined as engaging with the app at least once per month throughout the three-month assessment period. In the GCBT group, adherence criteria allowed participants to miss up to four sessions.

The adherence criterion for the app group (at least one interaction per month) was established to ensure a low barrier to participation, allowing for the inclusion of users with varying engagement patterns. This flexible approach acknowledges that individuals may benefit from the intervention at different paces while preventing the exclusion of those with irregular usage

### Sample size

The sample size calculation was based on a one-tailed Student *t*-test for differences between intervention arms (app and GCBT) and waiting list, considering an effect size (*d)* of 0.5, power (1- β) of 0.8, and significance level of 5%. The total calculated sample size was 100 (50 per comparison group). The *pwr* R package (version 1.3) was used for sample size calculation.

During the screening process, 868 individuals were invited or contacted to participate. Of these, 109 were included in the study, but 10 declined to participate after randomization, stating lack of availability to attend the weekly sessions or inability to access the app. The initial total sample size encompassed 99 participants (25 in the GCBT, 53 in the app-based intervention, and 21 in the WLC group). This high number of exclusions includes participants who did not answer phone calls, emails, or WhatsApp messages, as well as participants who were not included because they did not meet the inclusion/exclusion criteria. At the end of the study, 58 participants presented at least one entry in the assessment questionnaires (GCBT = 19; app-based intervention group = 28; WLC = 11). 41 participants had not completed any assessment questionnaire throughout their participation and were considered dropouts.

The recruitment and screening period lasted about a year, during which we encountered difficulties due to the high number of individuals not included for the reasons mentioned above. Consequently, we decided to close this stage prior to achieving the minimum sample size calculated. Additionally, we faced considerable challenges due to a high dropout rate, resulting in several assessment questionnaires remaining unanswered by participants initially included in the study.

### Statistical analysis

Numeric variables were summarized using median and interquartile ranges, and categorical variables were reported as absolute and relative frequencies. The *p*-values for the descriptive tables were calculated based on the Kruskal-Wallis rank sum test, Fisher's exact test or Pearson's chi-squared test, according to variable type and distribution.

In order to test differences in pre- and post-treatment changes (Δ) in PHQ-9, GAD-7 and UCLA-brief outcomes between the intervention groups and waiting list, we used one-tailed Student's *t*-tests and Mann-Whitney U tests, according to variable distribution. The *p*-values were adjusted based on Benjamini and Hochberg correction, also known as false discovery rate (FDR).^23^ The statistical significance level of 5% was used for all comparisons. Adverse effects were summarized based on its frequency and intensity.

In order to handle missing data, we excluded all subjects that presented less than two assessments (baseline assessment plus at least one follow-up) for any of the outcomes (PHQ-9, GAD-7 and UCLA-brief). For pre- and post-treatment analysis, we considered the latest assessment for each study participant.

All analyses were performed using the R programming language (version 4.4.1) running on Bash shell (version 5.2), Arch Linux (kernel version 6.10). Data visualizations were built using the *ggplot2* R package (version 3.5.1) and the *ggridges* R package (version 0.5.6).

## Results

### Sample characteristics

The sample characteristics of the 58 participants (intervention groups – GCBT and app-based intervention group; comparison group – WLC) are presented in Table 1. The sample predominantly comprised women (app: 86%; GCBT: 89%; WLC: 100%), subjects identified as white (app: 61%; GCBT: 63%; WLC: 82%), with median ages from 36 to 39, and relatively high income and education levels. There were no statistically significant differences in the distribution of these variables among groups, suggesting a homogeneity in demographic characteristics.

**Table 1:** Sample sociodemographic characteristics by treatment arm and waiting list (n = 58)

Characteristic		App N = 28	GCBT N = 19	WLC N = 11	p-value*
Age (in years), median (IQR)		39 (33 – 48)	37 (28 – 50)	36 (25 – 42)	0.31
Skin color, n (%)					0.51
	Non-white	11 (39)	7 (37)	2 (18)	
	White	17 (61)	12 (63)	9 (82)	
Sex, n (%)					0.55
	Female	24 (86)	17 (89)	11 (100)	
	Male	4 (14)	2 (11)	0 (0)	
Sexual orientation, n (%)					0.54
	Bisexual	2 (7.1)	1 (5.3)	2 (18)	
	Heterosexual	25 (89)	16 (84)	9 (82)	
	Homosexual	1 (3.6)	2 (11)	0 (0)	
Relationship status, n (%)					0.33
	Dating	2 (7.1)	3 (16)	1 (9.1)	
	Divorced	1 (3.6)	1 (5.3)	0 (0)	
	Married or stable union	17 (61)	7 (37)	3 (27)	
	Single	8 (29)	8 (42)	7 (64)	
	Having children, n (%)	15 (54)	8 (42)	4 (36)	0.56
Family income (in Brazilian reais), n (%)					0.60
	R$ 1.045,00 to R$ 3.135,00	10 (36)	8 (42)	3 (27)	
	R$ 3.135,00 to R$ 5.225,00	8 (29)	2 (11)	3 (27)	
	R$ 5.225,00 to R$ 15.675,00	9 (32)	6 (32)	4 (36)	
	Less than R$ 1045,00	1 (3.6)	2 (11)	0 (0)	
	Over R$ 15.675,00	0 (0)	1 (5.3)	1 (9.1)	
Education level, n (%)					0.51
	Completed high school	2 (7.1)	3 (16)	2 (18)	
	Completed undergraduate degree	12 (43)	7 (37)	2 (18)	
	Incomplete undergraduate degree	5 (18)	6 (32)	3 (27)	
	Post-graduate studies (incomplete or complete)	9 (32)	3 (16)	4 (36)	
Currently working, n (%)					0.47
	Retired	1 (3.6)	2 (11)	0 (0)	
	Unemployed	5 (18)	2 (11)	0 (0)	
	Studying	2 (7.1)	2 (11)	2 (18)	
	Yes, with a signed contract	7 (25)	7 (37)	2 (18)	
	Yes, I don't have a formal contract, but I am a civil servant	7 (25)	4 (21.3)	2 (18)	
	Yes, I don't have a formal employment contract, but I work informally/independently	6 (21)	2 (11)	5 (45)	

GCBT = group cognitive-behavioral therapy; IQR = interquartile range; WLC = waiting-list control.

* Kruskal-Wallis rank sum test; Fisher's exact test; Pearson's chi-squared test.

### Pre- and post-treatment changes

**Figure 2:**
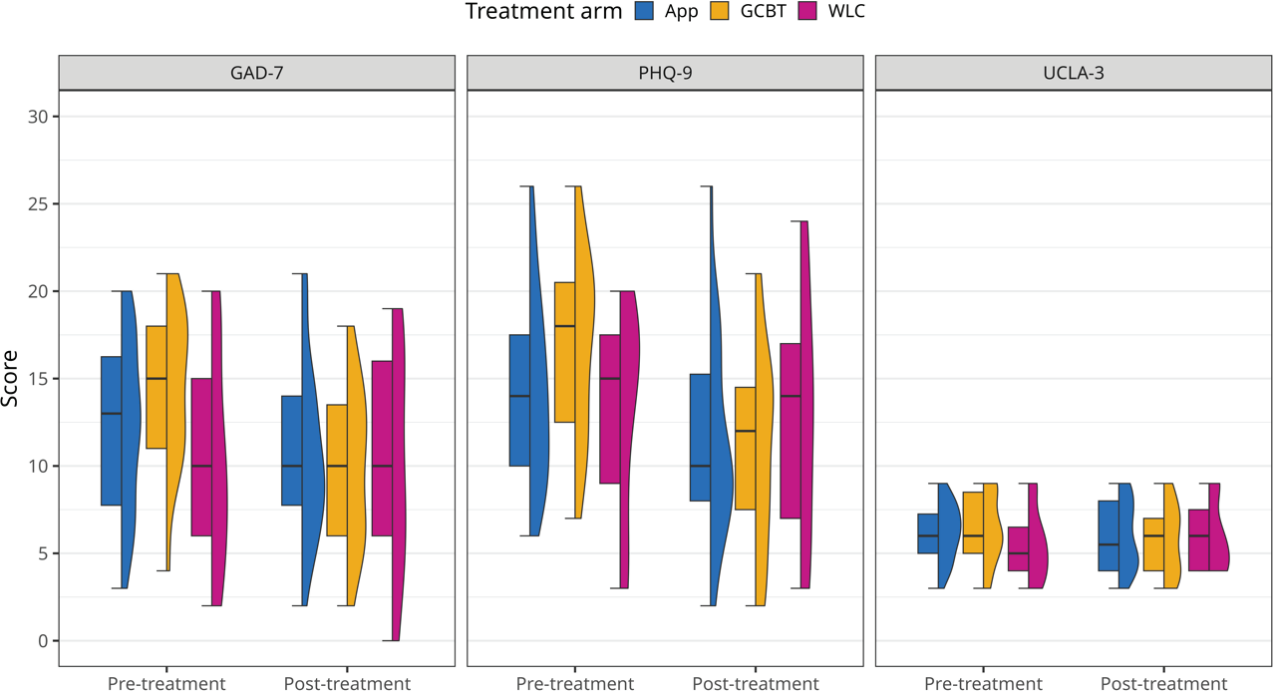
Distribution of GAD-7, PHQ-9, and UCLA-3 score before and after treatment by treatment arm. The violin plots display the kernel density estimation of the data, while the overlaid boxplots represent the median, interquartile range (IQR), and 1.5x IQR whiskers. Anxiety (GAD-7) and depressive (PHQ-9) symptoms decreased from pre- to post-treatment across all intervention groups. In contrast, distributions remained stable in the waiting list group, as well as for loneliness symptoms (UCLA-3). GCBT = group cognitive-behavioral therapy; IQR = interquartile range; PHQ-9 = Patient Health Questionnaire; GAD-7 = Generalized Anxiety Disorder 7-item; UCLA-3 = University of California, Los Angeles - Loneliness Scale; WLC = waiting-list control.

**Figure 3:**
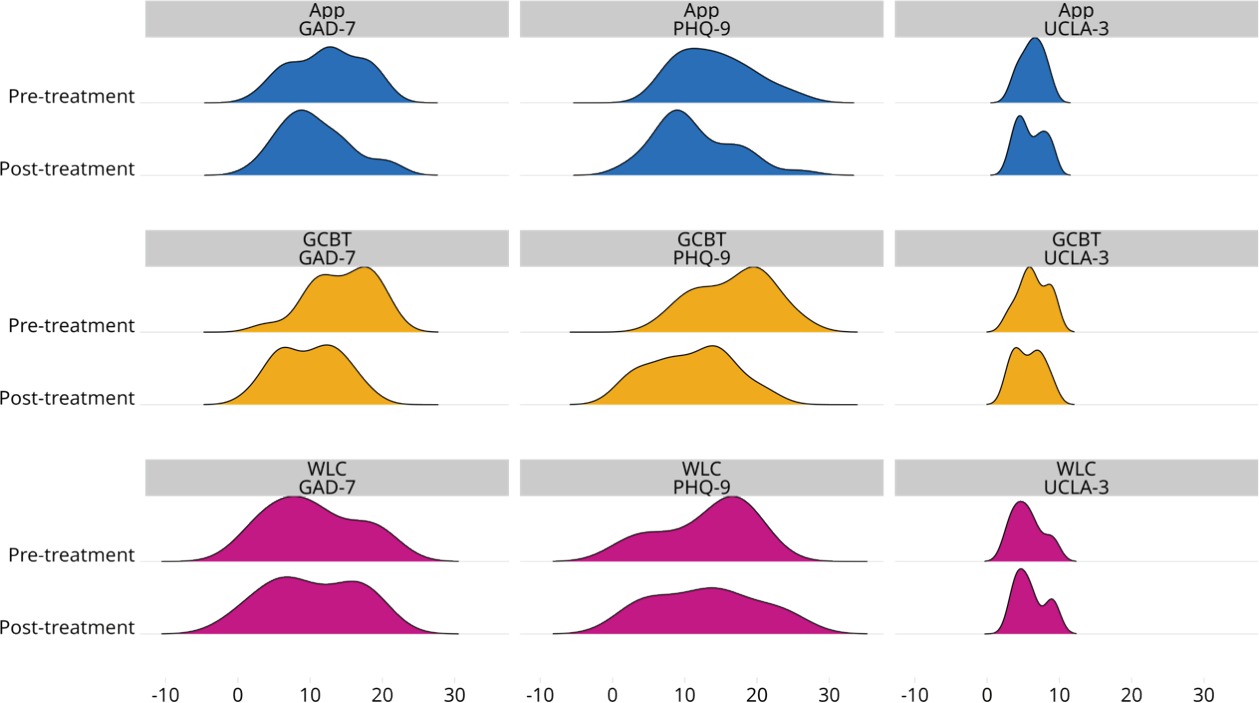
Density plots of GAD-7, PHQ-9, and UCLA-3 pre- and post-treatment scores stratified by treatment arm and waiting list. The *x*-axis is fixed in all visualizations to improve comparability. A discernible trend of the curves deviating to the left is evident, indicating a decline in symptomatology scores, particularly in the PHQ-9 and GAD-7 domains within the treatment arms. GCBT = group cognitive-behavioral therapy; PHQ-9 = Patient Health Questionnaire; GAD-7 = Generalized Anxiety Disorder 7-item; UCLA-3 = University of California, Los Angeles - Loneliness Scale; WLC = waiting-list control.

The analysis included 58 participants in total (32.8% GCBT; 48.3% app-based intervention group; 18.9% WLC). Table 2 presents pre- and post-treatment PHQ-9, GAD-7 and UCLA-3 scores for the participants in each treatment arm. The overall trend in symptom severity is particularly evident in anxiety- and depression-related outcomes. Since the study aimed to compare symptom reduction across three outcomes between online-based interventions and a waiting list control, the primary metric was the difference between pre- and post-treatment scores. The distributions of pre- and post-treatment data are available in Figure 2 and Figure 3.

**Table 2:** Depressive (PHQ-9), anxiety (GAD-7) and loneliness (UCLA-3) symptoms in pre- and post-treatment assessments stratified by treatment arm and waiting list (*n* = 58)

Outcome	Pre-treatment			Post-treatment		
	App N = 28	GCBT N = 19	WLC N = 11	App N = 28	GCBT N = 19	WLC N = 11
PHQ-9, Median (IQR)	14.0 (10.0–18.0)	18.0 (12.0–21.0)	15.0 (7.0–18.0)	10.0 (8.0–15.5)	12.0 (7.0–15.0)	14.0 (5.0–19.0)
GAD-7, Median (IQR)	13.0 (7.5–16.5)	15.0 (11.0–18.0)	10.0 (6.0–18.0)	10.0 (7.5–14.0)	10.0 (6.0–14.0)	10.0 (6.0–16.0)
UCLA-3, Median (IQR)	6.00 (5.00–7.50)	6.00 (5.00–9.00)	5.00 (4.00–7.00)	5.50 (4.00–8.00)	6.00 (4.00–7.00)	6.00 (4.00–9.00)

GCBT = group cognitive-behavioral therapy; IQR = interquartile range; PHQ-9 = Patient Health Questionnaire; GAD-7 = Generalized Anxiety Disorder 7-item; UCLA-3 = University of California, Los Angeles - Loneliness Scale; WLC = waiting-list control.

The results of these comparisons are available in Table 3. After correcting *p*-value for multiple comparisons, only GCBT demonstrated a significant post-treatment change in GAD-7 scores compared the waiting list (*t*(23.92) = 2.20, p = 0.019; p_adj_ = 0.038; Cohen's *d* = 0.81, 95%CI [0.17, ∞). The remaining comparisons were not statistically significant after *p*-value corrections.

**Table 3:** Results of comparisons between treatment arms (app and GCBT) and waiting list groups in depression (PHQ-9), anxiety (GAD-7) and loneliness (UCLA-3) outcomes regarding pre- and post-treatment differences (Δ). Significant corrected *p*-values are highlighted in bold

Outcome	Treatment arm	Statistic	DF	p-value	Adjusted *p*-value
GAD-7	App	0.812	22.9	0.213	0.213
GAD-7	GCBT	2.200	23.9	0.019	**0.038**
PHQ-9	App	0.867	16.4	0.199	0.199
PHQ-9	GCBT	1.900	15.3	0.038	0.076
UCLA-3	App	1.900	17.7	0.037	0.074
UCLA-3	GCBT	190.000	-	0.128*	0.128*

DF = degrees of freedom; GCBT = group cognitive-behavioral therapy; PHQ-9 = Patient Health Questionnaire; GAD-7 = Generalized Anxiety Disorder 7-item; UCLA-3 = University of California, Los Angeles - Loneliness Scale.

* Tested using Mann-Whitney U test. Adjusted *p*-values were corrected based on Benjamini and Hochberg method, known as false discovery rate (FDR) correction.^23^

### Treatment adherence

Among 47 participants included in any of the treatment arms (GCBT or app), 24 subjects (13 from GCBT [68.4%] and 11 from app-based intervention group [39.3%]) met the adherence criteria (less than five absences in GCBT and at least one interaction/month in the app), representing 51.1% of all participants who received an intervention.

Among the 11 subjects in the app-based intervention group, the average number of interactions in the first month was 142±220 (range: 17–783) interactions, 31.2±30.5 (range: 4–112) in the second month, and 15.4±17.2 (range: 1–53) in the third month. The average number of interactions in the app during the whole duration of the intervention (3 months) was 188±234 (range: 37–865).

### Adverse events

Regarding adverse events, the study's rigorous monitoring identified instances of suicidal ideation among participants, necessitating prompt intervention and clinical management. 22 participants exhibited suicidal ideation (a positive response on the last item of the PHQ-9 at any assessment). Only 1 participant was excluded due to high risk and need for hospitalization. The remaining individuals were contacted by the research team, underwent evaluation by experienced psychologists or psychiatrists in managing these symptoms, and were able to continue in the study after receiving guidance and appropriate management.

The overall incidence of adverse effects related to the interventions, evaluated by the Negative Effects Questionnaire, were presented in Supplementary Table S2, Table S3 and Figure S3. Analysis of adverse effects using NEQ revealed a significant occurrence of symptoms related to depression during the initial weeks, with a trend toward reduction over the course of treatment. Additionally, there was a notable frequency of unpleasant memories resurfacing throughout the study period in both intervention groups. Moreover, item 15 ("I did not always understand my treatment") showed a higher frequency of responses in the app-based intervention group compared to GCBT at week 4.

## Discussion

Our findings suggest a reduction in depressive and anxiety symptoms among the intervention groups compared to the WLC. However, while the GCBT intervention led to a statistically significant reduction in anxiety symptoms, the reduction in depressive symptoms did not reach statistical significance in either group. The observed changes in the app-based intervention group should be interpreted with caution, as they may reflect exploratory trends rather than definitive effects.

These results are consistent with the literature, which suggests a hierarchy among different types of digital interventions. The two intervention groups in this study were quite similar in terms of treatment duration, psychoeducation content, and applied techniques, differing only in the presence of a therapist in synchronous group sessions. A recent meta-analysis comparing face-to-face CBT, guided and unguided internet-based CBT (iCBT) showed that all formats were effective when compared to placebos. However, synchronous interventions were superior to guided asynchronous ones, which, in turn, were more effective than unguided asynchronous interventions.^24^ GCBT's significant reduction in anxiety symptoms, compared to the app intervention, warrants further exploration. This difference may be attributed to the role of therapist-led interactions, which provide personalized feedback, emotional support, and real-time adaptation of therapeutic strategies.^25,26^ In contrast, the app intervention may lack these dynamic elements, potentially limiting its effectiveness. Future improvements to the app design could focus on incorporating interactive features, such as AI-driven adaptive feedback or periodic therapist check-ins, to enhance engagement and therapeutic outcomes.

The emotional state of loneliness was also assessed in our sample. It is important to highlight that median scores around 6 were found, which emphasizes the high prevalence of loneliness among individuals with depressive symptoms. To assess this outcome, we used the UCLA-3 item Loneliness Scale, a widely recognized tool for quickly and reliably measuring perceived levels of loneliness, with individuals scoring between 6 and 9 on this scale classified as experiencing loneliness.^27^ There was little variation in loneliness scores throughout the study, which may be attributed to the lack of in-person contact, as both interventions were conducted online. Study arms lacked close human interaction, highlighting a potential limitation of digital interventions. These findings raise an important concern regarding the effectiveness of online-based treatments in addressing loneliness, a key symptom often accompanying depression. While digital interventions offer accessibility and scalability, their inability to foster meaningful human connection may limit their impact on social well-being.

Several meta-analyses have demonstrated varying effect sizes in the use of smartphone application interventions for depressive symptoms, ranging from small,^28-30^ moderate,^17,31-33^ to large effect sizes.^34^ A recent meta-analysis, published in 2023, comprised 13 studies evaluating 16 smartphone application interventions, totaling 1470 participants with moderate to severe depression, demonstrating a moderate effect size (SMD, 0.50; 95%CI, 0.40 to 0.61) with substantial heterogeneity (Q = 46.18; P < .001; I2 = 67.5%). This meta-analysis demonstrated that the applications produced yield a significant effect size when used both independently and as adjunct treatment to conventional therapies. However, the effect sizes were more pronounced in participants who were not receiving ongoing treatment.^17^ This finding assertion differs from previous studies and may be explained by the ceiling effect, where patients already undergoing treatment have a reduced potential for further improvement.^32^ In our sample, over a third of the participants were taking concomitant psychiatric medication (34.5%), and many did not meet the criteria for major depressive disorder, which may have potentially contributed to the mild variation in scores. Thus, we advocate for the role of applications integrated into a clinical context, as an adjunctive tool with professionals providing guidance and support.

Regarding adverse events, the study's rigorous monitoring identified instances of suicidal ideation among participants, necessitating prompt intervention and clinical management. However, the overall incidence of adverse effects related to the interventions was relatively low. Analysis of adverse effects using NEQ revealed a significant occurrence of sleep problems, stress, anxiety, and worries during the initial weeks, with a trend toward reduction over the course of treatment. Additionally, there was a notable frequency of unpleasant memories resurfacing throughout the study period in both intervention groups. Moreover, item 15 ("I did not always understand my treatment") showed a higher frequency of responses in the app-based intervention group compared to GCBT at week 4. Although this difference diminished over time, it suggests potential challenges in comprehending the app-based intervention. These findings highlight the need to address communication and comprehension difficulties, which may be more prevalent in asynchronous digital interventions lacking direct and continuous interaction with a therapist.

Our study has some limitations. The sample size for each intervention group was lower than initially estimated due to logistical and time constraints, limiting the strength of our findings and not allowing us to conduct subgroup analyses based on gender, age, or socioeconomic status. Exploring these aspects would have been valuable, as they could potentially influence patterns of usage and engagement with the application. Additionally, due to challenges in reaching the required sample size, we had to broaden the inclusion criteria, accepting patients with depressive symptoms who did not necessarily meet the criteria for major depressive disorder. Patients with mild or subclinical depressive symptoms tend to have lower symptom remission due to symptom heterogeneity, lower severity, and less engagement in treatment compared to those diagnosed with major depressive disorder.

The generalizability of our findings should be considered in light of the sample characteristics. The study predominantly included women, individuals who self-identified as white, and participants with relatively high income and education levels. This demographic profile may limit the applicability of the results to more diverse populations, particularly those with different socioeconomic and cultural backgrounds. Future research should aim to include more heterogeneous samples to enhance the external validity of these findings.

Additionally, we experienced a high dropout rate, particularly during and shortly after the screening phase. Throughout the study period, dropout rates were higher in the app-based intervention group, despite our efforts to implement recommendations from recent meta-analyses aimed at reducing these rates. For instance, providing human feedback and mood monitoring within the app were adherence-enhancing factors incorporated into our study.^29^ One of the factors that may have contributed to the low adherence was the fact that the app experienced technical issues during the participation of one of the groups, remaining offline for 4 weeks and may have affected user engagement. Similar randomized controlled trials (RCTs) conducted in Hispanic and Latino populations highlight the issue of dropout rates, reporting a participation rate of 50% from week 1 to week 4, which then declined sharply to 14% by the end of the 12-week period.^35^ The persistent challenge of maintaining long-term engagement, as highlighted in other studies focusing on Hispanic/Latino populations,^36^ underscores the importance of addressing low engagement as a significant factor in future research.

The small final sample size and high dropout rate represent important limitations of this study, as they may compromise the generalizability of the findings. High attrition may introduce selection bias, as participants who completed the study might differ systematically from those who dropped out. Future studies should implement strategies to mitigate these challenges, such as enhancing participant engagement through more personalized follow-ups, offering incentives for study completion, and employing intention-to-treat analyses to account for missing data. Additionally, ensuring an adequate initial sample size and using adaptive trial designs may help counterbalance potential dropout effects.

This treatment model, which necessitates active engagement from the patient, may pose a challenge for the Brazilian population. However, delivering treatment through a web application provides tools similar to those found in in-person cognitive behavioral therapy at a significantly reduced cost. An interesting find from this study is the absence of observed deterioration over time in either format. This information should be highlighted to draw clinicians’ attention to the potency of these apps as valuable tools for maintaining treatment outcomes. Additionally, it signals a direction for the academic community, emphasizing the increasing role of these apps as a crucial link connecting clinicians with their patients. Nevertheless, we believe that digital interventions, particularly those delivered through apps, hold promise as an attractive future strategy both for patients undergoing therapy who can benefit from a support tool between sessions and for individuals who have previously undergone therapy and are in a stable phase, focusing on maintenance. Future studies are essential to clarify how these interventions can effectively benefit such populations.

## Conclusion

In conclusion, this study has provided valuable insights into the effectiveness of mobile application-based intervention and GCBT in treating depressive and anxiety symptoms. Our findings indicate that both treatments led to a reduction in symptoms over time, with statistically significant results observed only for anxiety symptoms in the GCBT group. Additionally, they highlight the feasibility and effectiveness of mobile application-based interventions as an accessible and convenient option, particularly when integrated into a clinical context and provided with professional guidance. Further exploration and refinement of these therapeutic approaches have the potential to not only enhance the benefits of treating common mental disorders but also to reduce the treatment gap, thereby promoting better mental health and quality of life for patients.

## supplementary material



## References

[1] McGrath JJ, Al-Hamzawi A, Alonso J, Altwaijri Y, Andrade LH, Bromet EJ (2023). Age of onset and cumulative risk of mental disorders: a cross-national analysis of population surveys from 29 countries. Lancet Psychiatry.

[2] Patel V, Chisholm D, Parikh R, Charlson FJ, Degenhardt L, Dua T (2016). Addressing the burden of mental, neurological, and substance use disorders: key messages from Disease Control Priorities, 3rd edition. Lancet.

[3] GBD 2019 Mental Disorders Collaborators (2022). Global, regional, and national burden of 12 mental disorders in 204 countries and territories, 1990-2019: a systematic analysis for the Global Burden of Disease Study 2019. Lancet Psychiatry.

[4] Santomauro DF, Mantilla Herrera AM, Shadid J, Zheng P, Ashbaugh C, Pigott DM (2021). Global prevalence and burden of depressive and anxiety disorders in 204 countries and territories in 2020 due to the COVID-19 pandemic. Lancet.

[5] Moitra M, Santomauro D, Collins PY, Vos T, Whiteford H, Saxena S (2022). The global gap in treatment coverage for major depressive disorder in 84 countries from 2000-2019: A systematic review and Bayesian meta-regression analysis. PLoS Med.

[6] Evans-Lacko S, Aguilar-Gaxiola S, Al-Hamzawi A, Alonso J, Benjet C, Bruffaerts R (2018). Socio-economic variations in the mental health treatment gap for people with anxiety, mood, and substance use disorders: results from the WHO World Mental Health (WMH) surveys. Psychol Med.

[7] Chisholm D, Sweeny K, Sheehan P, Rasmussen B, Smit F, Cuijpers P (2016). Scaling-up treatment of depression and anxiety: a global return on investment analysis. Lancet Psychiatry.

[8] Rugulies R (2002). Depression as a predictor for coronary heart disease. a review and meta-analysis. Am J Prev Med.

[9] Jacobson NC, Newman MG (2017). Anxiety and depression as bidirectional risk factors for one another: A meta-analysis of longitudinal studies. Psychol Bull.

[10] Antonelli-Salgado T, Monteiro GMC, Marcon G, Roza TH, Zimerman A, Hoffmann MS (2021). Loneliness, but not social distancing, is associated with the incidence of suicidal ideation during the COVID-19 outbreak: a longitudinal study. J Affect Disord.

[11] Chen Z, Song X, Lee TMC, Zhang R (2023). The robust reciprocal relationship between loneliness and depressive symptoms among the general population: Evidence from a quantitative analysis of 37 studies. J Affect Disord.

[12] Pinquart M, Duberstein PR (2010). Depression and cancer mortality: a meta-analysis. Psychol Med.

[13] Howarth J (2021). How many people own smartphones? (2024-2029) [Internet]. Exploding Topics.

[14] Andersson G, Cuijpers P (2009). Internet-based and other computerized psychological treatments for adult depression: a meta-analysis. Cogn Behav Ther.

[15] Marshall JM, Dunstan DA, Bartik W (2020). Apps with maps—anxiety and depression mobile apps with evidence-based frameworks: systematic search of major app stores. JMIR Mental Health.

[16] Office of the Commissioner (2024). FDA Roundup: April 2, 2024 [Internet].

[17] Bae H, Shin H, Ji H-G, Kwon JS, Kim H, Hur J-W (2023). App-based interventions for moderate to severe depression: a systematic review and meta-analysis. JAMA Netw Open.

[18] Piaggio G, Elbourne DR, Pocock SJ, Evans SJW, Altman DG, CONSORT Group (2012). Reporting of noninferiority and equivalence randomized trials: extension of the CONSORT 2010 statement. JAMA.

[19] Amorim P (2000). Mini International Neuropsychiatric Interview (MINI): validation of a short structured diagnostic psychiatric interview. Braz J Psychiatry.

[20] Kroenke K, Spitzer RL, Williams JB (2001). The PHQ-9: validity of a brief depression severity measure. J Gen Intern Med.

[21] Braga DT, Vivan AS, Passos IC (2024). Vencendo a Depressão: Manual de Terapia Cognitivo-comportamental para Pacientes e Terapeutas.

[22] Rozental A, Kottorp A, Forsström D, Månsson K, Boettcher J, Andersson G (2019). The Negative Effects Questionnaire: psychometric properties of an instrument for assessing negative effects in psychological treatments. Behav Cogn Psychother.

[23] Benjamini Y, Hochberg Y (1995). Controlling the false discovery rate: A practical and powerful approach to multiple testing. J R Stat Soc Series B Stat Methodol.

[24] Zhang W, Yang W, Ruan H, Gao J, Wang Z (2023). Comparison of internet-based and face-to-face cognitive behavioral therapy for obsessive-compulsive disorder: a systematic review and network meta-analysis. J Psychiatr Res.

[25] Conrad A, Roth WT (2007). Muscle relaxation therapy for anxiety disorders: it works but how?. J Anxiety Disord.

[26] Koszycki D, Benger M, Shlik J, Bradwejn J (2007). Randomized trial of a meditation-based stress reduction program and cognitive behavior therapy in generalized social anxiety disorder. Behav Res Ther.

[27] Hughes ME, Waite LJ, Hawkley LC, Cacioppo JT (2004). A short scale for measuring loneliness in large surveys: results from two population-based studies. Res Aging.

[28] Wu A, Scult MA, Barnes ED, Betancourt JA, Falk A, Gunning FM (2021). Smartphone apps for depression and anxiety: a systematic review and meta-analysis of techniques to increase engagement. NPJ Digit Med.

[29] Linardon J, Cuijpers P, Carlbring P, Messer M, Fuller-Tyszkiewicz M (2019). The efficacy of app-supported smartphone interventions for mental health problems: a meta-analysis of randomized controlled trials. World Psychiatry.

[30] Park C, Zhu J, Ho Chun Man R, Rosenblat JD, Iacobucci M, Gill H (2020). Smartphone applications for the treatment of depressive symptoms: A meta-analysis and qualitative review. Ann Clin Psychiatry.

[31] Firth J, Torous J, Nicholas J, Carney R, Pratap A, Rosenbaum S (2017). The efficacy of smartphone-based mental health interventions for depressive symptoms: a meta-analysis of randomized controlled trials. World Psychiatry.

[32] Weisel KK, Fuhrmann LM, Berking M, Baumeister H, Cuijpers P, Ebert DD (2019). Standalone smartphone apps for mental health-a systematic review and meta-analysis. NPJ Digit Med.

[33] Serrano-Ripoll MJ, Zamanillo-Campos R, Fiol-DeRoque MA, Castro A, Ricci-Cabello I (2022). Impact of smartphone app-based psychological interventions for reducing depressive symptoms in people with depression: systematic literature review and meta-analysis of randomized controlled trials. JMIR Mhealth Uhealth.

[34] Josephine K, Josefine L, Philipp D, David E, Harald B (2017). Internet- and mobile-based depression interventions for people with diagnosed depression: a systematic review and meta-analysis. J Affect Disord.

[35] Pratap A, Renn BN, Volponi J, Mooney SD, Gazzaley A, Arean PA (2018). Using mobile apps to assess and treat depression in Hispanic and Latino populations: fully remote randomized clinical trial. J Med Internet Res.

[36] Brown G, Marshall M, Bower P, Woodham A, Waheed W (2014). Barriers to recruiting ethnic minorities to mental health research: a systematic review. Int J Methods Psychiatr Res.

