# Supplementary material for: Digital interventions for depressive symptoms: a randomized clinical trial
Source: Trends Psychiatry Psychother. 2025 Nov 5;47:e20241006. doi: 10.47626/2237-6089-2024-1006 (PMC12956145; doi:10.47626/2237-6089-2024-1006)
Supplement: Supplementary file 1 [file 2238-0019-trends-47-e20241006-suppl01.pdf]

**Figure S1** - Thrive app screens: A) App icon in the app store. B) App startup screen. C) Psychoeducation screen. D) Monitoring screen: mood assessment.

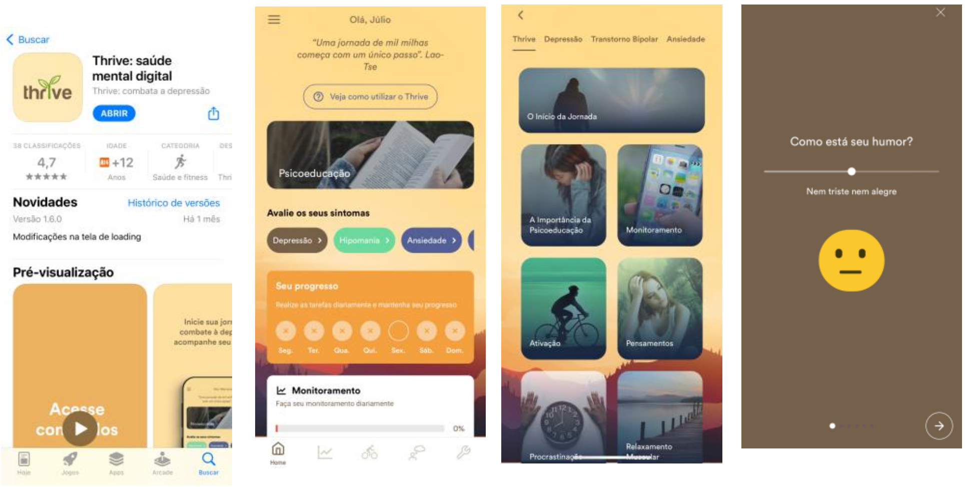


**Figure S2** - A) Monitoring screen: anxiety symptom assessment. B) Monitoring screen: sleep hours assessment. C) Behavioral activation screen: activity selection. D) Thought recording screen: description of the triggering event.

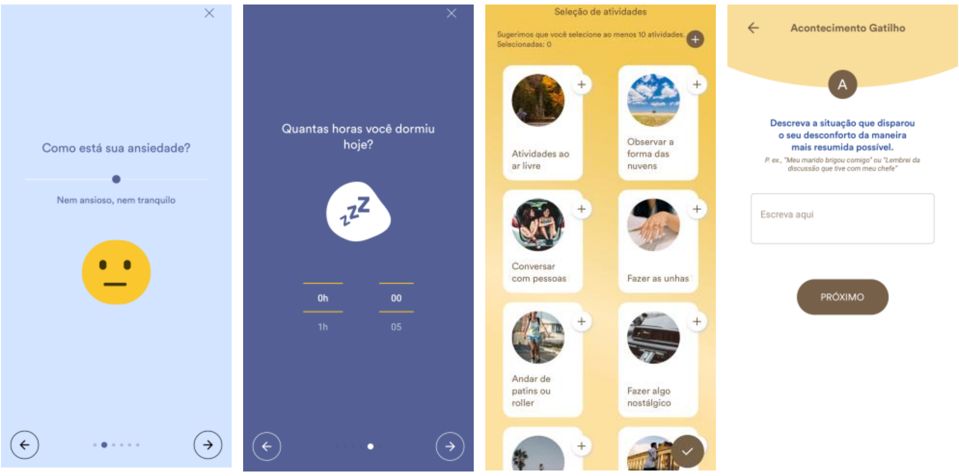

http://doi.org/10.47626/2237-6089-2024-1006


**Figure S3** - Frequency of adverse effects in each assessment during treatment period grouped by treatment arm. Vertical line indicates 50% frequency of reported effect. The item descriptions are available at Table S1.

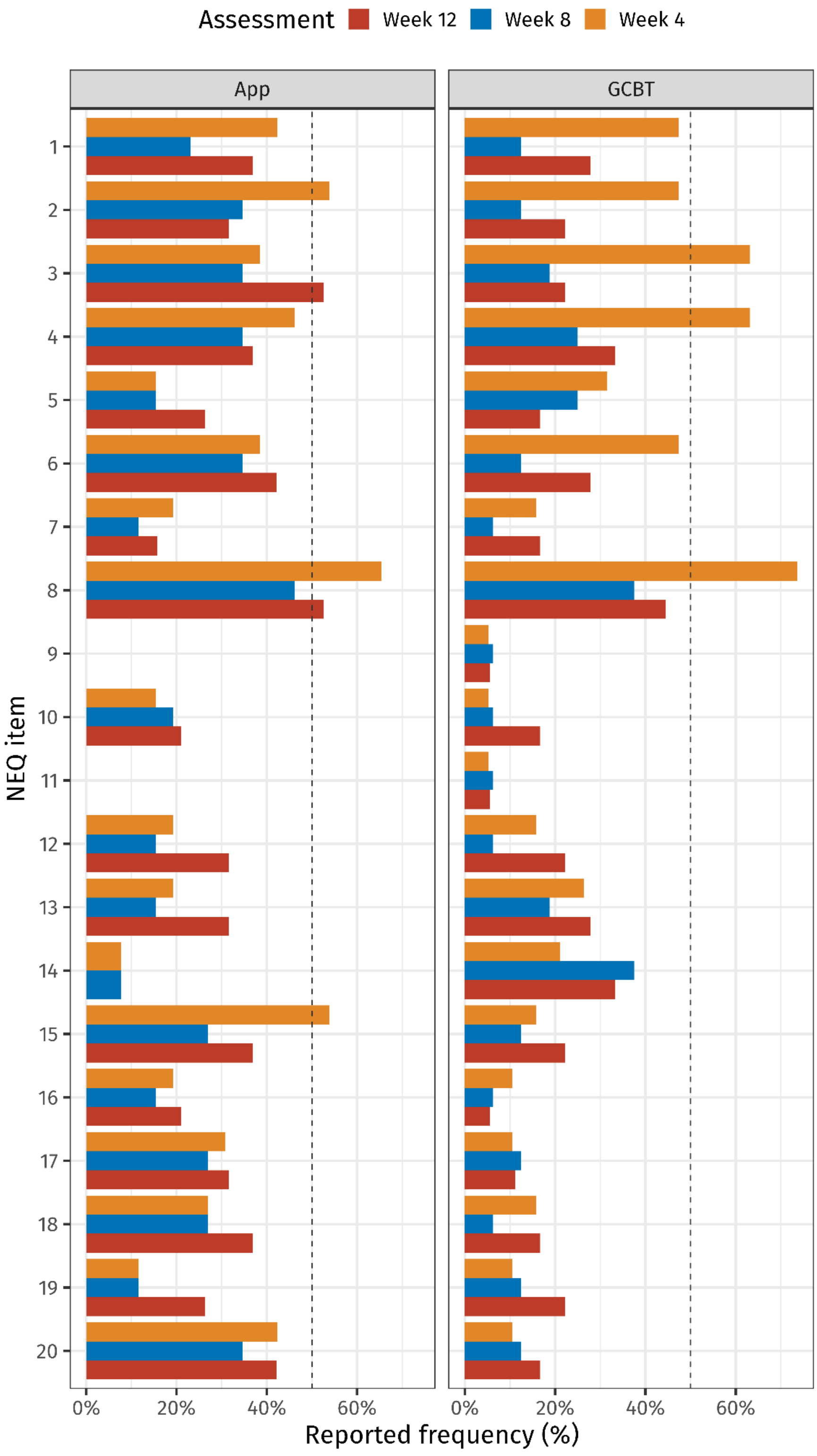

http://doi.org/10.47626/2237-6089-2024-1006


**Table S1 -** Reported adverse effects and their corresponding items on the NEQ (administered to participants at weeks 4, 8, and 12 of the intervention)

| NEQ Item | Adverse effect |
|---|---|
| 1 | I had more problems with my sleep |
| 2 | I felt like I was under more stress |
| 3 | I experienced more anxiety |
| 4 | I felt more worried |
| 5 | I experienced more hopelessness |
| 6 | I experienced more unpleasant feelings |
| 7 | I felt that the issue I was looking for help with got worse |
| 8 | Unpleasant memories resurfaced |
| 9 | I became afraid that other people would find out about my treatment |
| 10 | I got thoughts that it would be better if I did not exist anymore and that I should take my own life |
| 11 | I started feeling ashamed in front of other people because I was having treatment |
| 12 | I stopped thinking that things could get better |
| 13 | I started thinking that the issue I was seeking help for could not be made any better |
| 14 | I think that I have developed a dependency on my treatment |
| 15 | I did not always understand my treatment |
| 16 | I did not always understand my therapist |
| 17 | I did not have confidence in my treatment |
| 18 | I felt that the treatment did not produce any results |
| 19 | I felt that my expectations for the therapist were not fulfilled |
| 20 | I felt that the treatment was not motivating |

**Table S2 -** Frequency of negative effects in each assessment stratified by treatment arm based on Negative Effects Questionnaire (*n* = 47)

| **NEQ item** | **Week 4** | | **Week 8** | | **Week 12** | |
|---|---|---|---|---|---|---|
| | **App** N = 26[1] | **GCBT** N = 19[1] | **App** N = 26[1] | **GCBT** N = 16[1] | **App** N = 19[1] | **GCBT** N = 18[1] |
| Item 01 | 11 (44%) | 9 (47%) | 6 (24%) | 2 (13%) | 7 (39%) | 5 (29%) |
| Missing | 1 | 0 | 1 | 1 | 1 | 1 |
| Item 02 | 14 (56%) | 9 (47%) | 9 (38%) | 2 (13%) | 6 (33%) | 4 (24%) |
| Missing | 1 | 0 | 2 | 1 | 1 | 1 |
| Item 03 | 10 (40%) | 12 (63%) | 9 (38%) | 3 (20%) | 10 (56%) | 4 (24%) |
| Missing | 1 | 0 | 2 | 1 | 1 | 1 |
| Item 04 | 12 (48%) | 12 (63%) | 9 (38%) | 4 (27%) | 7 (39%) | 6 (35%) |
| Missing | 1 | 0 | 2 | 1 | 1 | 1 |
| Item 05 | 4 (16%) | 6 (32%) | 4 (17%) | 4 (27%) | 5 (28%) | 3 (18%) |
| Missing | 1 | 0 | 2 | 1 | 1 | 1 |
| Item 06 | 10 (40%) | 9 (47%) | 9 (38%) | 2 (13%) | 8 (44%) | 5 (29%) |
| Missing | 1 | 0 | 2 | 1 | 1 | 1 |
| Item 07 | 5 (20%) | 3 (16%) | 3 (13%) | 1 (6.7%) | 3 (17%) | 3 (18%) |
| Missing | 1 | 0 | 2 | 1 | 1 | 1 |
| Item 08 | 17 (68%) | 14 (74%) | 12 (50%) | 6 (40%) | 10 (56%) | 8 (47%) |
| Missing | 1 | 0 | 2 | 1 | 1 | 1 |
| Item 09 | 0 (0%) | 1 (5.3%) | 0 (0%) | 1 (6.7%) | 0 (0%) | 1 (5.9%) |
| Missing | 1 | 0 | 2 | 1 | 1 | 1 |
| Item 10 | 4 (16%) | 1 (5.3%) | 5 (21%) | 1 (6.7%) | 4 (22%) | 3 (18%) |
| Missing | 1 | 0 | 2 | 1 | 1 | 1 |
| Item 11 | 0 (0%) | 1 (5.3%) | 0 (0%) | 1 (6.7%) | 0 (0%) | 1 (5.9%) |
| Missing | 1 | 0 | 2 | 1 | 1 | 1 |
| Item 12 | 5 (20%) | 3 (16%) | 4 (17%) | 1 (6.7%) | 6 (33%) | 4 (24%) |
| Missing | 1 | 0 | 2 | 1 | 1 | 1 |
| Item 13 | 5 (20%) | 5 (26%) | 4 (17%) | 3 (20%) | 6 (33%) | 5 (29%) |
| Missing | 1 | 0 | 2 | 1 | 1 | 1 |
| Item 14 | 2 (8.0%) | 4 (21%) | 2 (8.3%) | 6 (40%) | 0 (0%) | 6 (35%) |
| Missing | 1 | 0 | 2 | 1 | 1 | 1 |
| Item 15 | 14 (56%) | 3 (16%) | 7 (29%) | 2 (13%) | 7 (39%) | 4 (24%) |
| Missing | 1 | 0 | 2 | 1 | 1 | 1 |
| Item 16 | 5 (20%) | 2 (11%) | 4 (17%) | 1 (6.7%) | 4 (22%) | 1 (5.9%) |
| Missing | 1 | 0 | 2 | 1 | 1 | 1 |
| Item 17 | 8 (32%) | 2 (11%) | 7 (29%) | 2 (13%) | 6 (33%) | 2 (12%) |
| Missing | 1 | 0 | 2 | 1 | 1 | 1 |
| Item 18 | 7 (28%) | 3 (16%) | 7 (29%) | 1 (6.7%) | 7 (39%) | 3 (18%) |
| Missing | 1 | 0 | 2 | 1 | 1 | 1 |
| Item 19 | 3 (12%) | 2 (11%) | 3 (13%) | 2 (13%) | 5 (28%) | 4 (24%) |
| Missing | 1 | 0 | 2 | 1 | 1 | 1 |
| Item 20 | 11 (44%) | 2 (11%) | 9 (38%) | 2 (13%) | 8 (44%) | 3 (18%) |
| Missing | 1 | 0 | 2 | 1 | 1 | 1 |

[1]n (%). Note: Item descriptions are available in Table S1.

http://doi.org/10.47626/2237-6089-2024-1006


**Table S3 -** Frequency of negative effects intensity reported by patients in the Negative Effects Questionnaire for each assessment stratified by treatment arm ($n$ = 47)

| NEQ Item | Week 4 | | Week 8 | | Week 12 | |
|---|---|---|---|---|---|---|
| | **App** N = 26* | **GCBT** N = 19* | **App** N = 26* | **GCBT** N = 16* | **App** N = 19* | **GCBT** N = 18* |
| Item 01 | | | | | | |
| I was not affected | 0 (0%) | 1 (11%) | 0 (0%) | 0 (0%) | 1 (14%) | 0 (0%) |
| Slightly | 3 (27%) | 2 (22%) | 3 (60%) | 1 (50%) | 1 (14%) | 1 (20%) |
| Moderately | 3 (27%) | 0 (0%) | 0 (0%) | 1 (50%) | 2 (29%) | 1 (20%) |
| Very | 4 (36%) | 4 (44%) | 1 (20%) | 0 (0%) | 3 (43%) | 3 (60%) |
| Extremely | 1 (9.1%) | 2 (22%) | 1 (20%) | 0 (0%) | 0 (0%) | 0 (0%) |
| N/A | 15 | 10 | 21 | 14 | 12 | 13 |
| Item 02 | | | | | | |
| I was not affected | 1 (7.1%) | 0 (0%) | 0 (0%) | 0 (0%) | 0 (0%) | 0 (0%) |
| Slightly | 2 (14%) | 1 (11%) | 2 (22%) | 0 (0%) | 4 (67%) | 0 (0%) |
| Moderately | 5 (36%) | 4 (44%) | 6 (67%) | 1 (50%) | 1 (17%) | 2 (50%) |
| Very | 5 (36%) | 3 (33%) | 1 (11%) | 1 (50%) | 1 (17%) | 2 (50%) |
| Extremely | 1 (7.1%) | 1 (11%) | 0 (0%) | 0 (0%) | 0 (0%) | 0 (0%) |
| N/A | 12 | 10 | 17 | 14 | 13 | 14 |
| Item 03 | | | | | | |
| I was not affected | 1 (10%) | 2 (17%) | 0 (0%) | 1 (33%) | 0 (0%) | 0 (0%) |
| Slightly | 3 (30%) | 0 (0%) | 2 (22%) | 0 (0%) | 5 (50%) | 1 (25%) |
| Moderately | 2 (20%) | 4 (33%) | 5 (56%) | 0 (0%) | 4 (40%) | 2 (50%) |
| Very | 3 (30%) | 4 (33%) | 0 (0%) | 2 (67%) | 1 (10%) | 1 (25%) |
| Extremely | 1 (10%) | 2 (17%) | 2 (22%) | 0 (0%) | 0 (0%) | 0 (0%) |
| N/A | 16 | 7 | 17 | 13 | 9 | 14 |
| Item 04 | | | | | | |
| I was not affected | 0 (0%) | 0 (0%) | 0 (0%) | 0 (0%) | 0 (0%) | 0 (0%) |
| Slightly | 5 (42%) | 1 (8.3%) | 3 (38%) | 0 (0%) | 2 (29%) | 0 (0%) |
| Moderately | 3 (25%) | 4 (33%) | 3 (38%) | 2 (50%) | 1 (14%) | 4 (67%) |
| Very | 4 (33%) | 6 (50%) | 1 (13%) | 2 (50%) | 4 (57%) | 2 (33%) |
| Extremely | 0 (0%) | 1 (8.3%) | 1 (13%) | 0 (0%) | 0 (0%) | 0 (0%) |
| N/A | 14 | 7 | 18 | 12 | 12 | 12 |
| Item 05 | | | | | | |
| I was not affected | 0 (0%) | 0 (0%) | 0 (0%) | 0 (0%) | 0 (0%) | 0 (0%) |
| Slightly | 1 (25%) | 1 (17%) | 1 (25%) | 1 (25%) | 1 (20%) | 0 (0%) |
| Moderately | 2 (50%) | 2 (33%) | 2 (50%) | 1 (25%) | 2 (40%) | 2 (67%) |
| Very | 1 (25%) | 3 (50%) | 0 (0%) | 1 (25%) | 2 (40%) | 1 (33%) |
| Extremely | 0 (0%) | 0 (0%) | 1 (25%) | 1 (25%) | 0 (0%) | 0 (0%) |
| N/A | 22 | 13 | 22 | 12 | 14 | 15 |
| Item 06 | | | | | | |
| I was not affected | 0 (0%) | 0 (0%) | 0 (0%) | 0 (0%) | 0 (0%) | 0 (0%) |
| Slightly | 4 (40%) | 1 (11%) | 3 (38%) | 0 (0%) | 1 (13%) | 1 (20%) |
| Moderately | 2 (20%) | 4 (44%) | 3 (38%) | 1 (50%) | 3 (38%) | 0 (0%) |
| Very | 4 (40%) | 4 (44%) | 2 (25%) | 1 (50%) | 4 (50%) | 4 (80%) |
| Extremely | 0 (0%) | 0 (0%) | 0 (0%) | 0 (0%) | 0 (0%) | 0 (0%) |
| N/A | 16 | 10 | 18 | 14 | 11 | 13 |
| Item 07 | | | | | | |
| I was not affected | 1 (20%) | 0 (0%) | 1 (33%) | 0 (0%) | 0 (0%) | 0 (0%) |
| Slightly | 1 (20%) | 0 (0%) | 0 (0%) | 0 (0%) | 1 (33%) | 1 (33%) |
| Moderately | 1 (20%) | 1 (33%) | 0 (0%) | 0 (0%) | 2 (67%) | 0 (0%) |
| Very | 2 (40%) | 2 (67%) | 1 (33%) | 1 (100%) | 0 (0%) | 2 (67%) |
| Extremely | 0 (0%) | 0 (0%) | 1 (33%) | 0 (0%) | 0 (0%) | 0 (0%) |
| N/A | 21 | 16 | 23 | 15 | 16 | 15 |
| Item 08 | | | | | | |
| I was not affected | 0 (0%) | 0 (0%) | 1 (8.3%) | 1 (17%) | 0 (0%) | 0 (0%) |
| Slightly | 7 (41%) | 4 (29%) | 2 (17%) | 3 (50%) | 1 (10%) | 2 (25%) |
| Moderately | 8 (47%) | 6 (43%) | 6 (50%) | 2 (33%) | 5 (50%) | 3 (38%) |
| Very | 2 (12%) | 3 (21%) | 1 (8.3%) | 0 (0%) | 4 (40%) | 3 (38%) |
| Extremely | 0 (0%) | 1 (7.1%) | 2 (17%) | 0 (0%) | 0 (0%) | 0 (0%) |

| NEQ Item | Week 4 | | Week 8 | | Week 12 | |
|---|---|---|---|---|---|---|
| | **App** N = 26* | **GCBT** N = 19* | **App** N = 26* | **GCBT** N = 16* | **App** N = 19* | **GCBT** N = 18* |
| N/A | 9 | 5 | 14 | 10 | 9 | 10 |
| Item 09 | | | | | | |
| I was not affected | 0 (NA%) | 0 (0%) | 0 (NA%) | 0 (0%) | 0 (NA%) | 0 (0%) |
| Slightly | 0 (NA%) | 0 (0%) | 0 (NA%) | 0 (0%) | 0 (NA%) | 0 (0%) |
| Moderately | 0 (NA%) | 0 (0%) | 0 (NA%) | 0 (0%) | 0 (NA%) | 0 (0%) |
| Very | 0 (NA%) | 1 (100%) | 0 (NA%) | 1 (100%) | 0 (NA%) | 1 (100%) |
| Extremely | 0 (NA%) | 0 (0%) | 0 (NA%) | 0 (0%) | 0 (NA%) | 0 (0%) |
| N/A | 26 | 18 | 26 | 15 | 19 | 17 |
| Item 10 | | | | | | |
| I was not affected | 0 (0%) | 0 (0%) | 0 (0%) | 0 (0%) | 0 (0%) | 0 (0%) |
| Slightly | 1 (25%) | 0 (0%) | 2 (40%) | 0 (0%) | 0 (0%) | 1 (33%) |
| Moderately | 2 (50%) | 1 (100%) | 0 (0%) | 0 (0%) | 2 (50%) | 1 (33%) |
| Very | 1 (25%) | 0 (0%) | 0 (0%) | 1 (100%) | 2 (50%) | 1 (33%) |
| Extremely | 0 (0%) | 0 (0%) | 3 (60%) | 0 (0%) | 0 (0%) | 0 (0%) |
| N/A | 22 | 18 | 21 | 15 | 15 | 15 |
| Item 11 | | | | | | |
| I was not affected | 0 (NA%) | 0 (0%) | 0 (NA%) | 0 (0%) | 0 (NA%) | 0 (0%) |
| Slightly | 0 (NA%) | 1 (100%) | 0 (NA%) | 0 (0%) | 0 (NA%) | 0 (0%) |
| Moderately | 0 (NA%) | 0 (0%) | 0 (NA%) | 1 (100%) | 0 (NA%) | 1 (100%) |
| Very | 0 (NA%) | 0 (0%) | 0 (NA%) | 0 (0%) | 0 (NA%) | 0 (0%) |
| Extremely | 0 (NA%) | 0 (0%) | 0 (NA%) | 0 (0%) | 0 (NA%) | 0 (0%) |
| N/A | 26 | 18 | 26 | 15 | 19 | 17 |
| Item 12 | | | | | | |
| I was not affected | 1 (20%) | 0 (0%) | 0 (0%) | 0 (0%) | 0 (0%) | 0 (0%) |
| Slightly | 2 (40%) | 0 (0%) | 0 (0%) | 0 (0%) | 1 (17%) | 1 (25%) |
| Moderately | 1 (20%) | 1 (33%) | 1 (25%) | 1 (100%) | 4 (67%) | 0 (0%) |
| Very | 1 (20%) | 2 (67%) | 3 (75%) | 0 (0%) | 1 (17%) | 3 (75%) |
| Extremely | 0 (0%) | 0 (0%) | 0 (0%) | 0 (0%) | 0 (0%) | 0 (0%) |
| N/A | 21 | 16 | 22 | 15 | 13 | 14 |
| Item 13 | | | | | | |
| I was not affected | 0 (0%) | 1 (20%) | 1 (25%) | 1 (33%) | 0 (0%) | 0 (0%) |
| Slightly | 0 (0%) | 1 (20%) | 0 (0%) | 0 (0%) | 1 (17%) | 1 (20%) |
| Moderately | 5 (100%) | 2 (40%) | 1 (25%) | 2 (67%) | 3 (50%) | 2 (40%) |
| Very | 0 (0%) | 1 (20%) | 1 (25%) | 0 (0%) | 2 (33%) | 2 (40%) |
| Extremely | 0 (0%) | 0 (0%) | 1 (25%) | 0 (0%) | 0 (0%) | 0 (0%) |
| N/A | 21 | 14 | 22 | 13 | 13 | 13 |
| Item 14 | | | | | | |
| I was not affected | 0 (0%) | 0 (0%) | 0 (0%) | 2 (33%) | 0 (NA%) | 0 (0%) |
| Slightly | 0 (0%) | 2 (50%) | 0 (0%) | 0 (0%) | 0 (NA%) | 2 (33%) |
| Moderately | 0 (0%) | 2 (50%) | 0 (0%) | 4 (67%) | 0 (NA%) | 3 (50%) |
| Very | 2 (100%) | 0 (0%) | 1 (50%) | 0 (0%) | 0 (NA%) | 1 (17%) |
| Extremely | 0 (0%) | 0 (0%) | 1 (50%) | 0 (0%) | 0 (NA%) | 0 (0%) |
| N/A | 24 | 15 | 24 | 10 | 19 | 12 |
| Item 15 | | | | | | |
| I was not affected | 5 (36%) | 1 (33%) | 0 (0%) | 0 (0%) | 2 (29%) | 0 (0%) |
| Slightly | 5 (36%) | 0 (0%) | 2 (29%) | 2 (100%) | 3 (43%) | 2 (50%) |
| Moderately | 3 (21%) | 2 (67%) | 4 (57%) | 0 (0%) | 1 (14%) | 2 (50%) |
| Very | 1 (7.1%) | 0 (0%) | 1 (14%) | 0 (0%) | 1 (14%) | 0 (0%) |
| Extremely | 0 (0%) | 0 (0%) | 0 (0%) | 0 (0%) | 0 (0%) | 0 (0%) |
| N/A | 12 | 16 | 19 | 14 | 12 | 14 |
| Item 16 | | | | | | |
| I was not affected | 1 (20%) | 0 (0%) | 0 (0%) | 0 (0%) | 1 (25%) | 0 (0%) |
| Slightly | 2 (40%) | 1 (50%) | 2 (50%) | 0 (0%) | 0 (0%) | 1 (100%) |
| Moderately | 2 (40%) | 1 (50%) | 1 (25%) | 0 (0%) | 2 (50%) | 0 (0%) |
| Very | 0 (0%) | 0 (0%) | 1 (25%) | 1 (100%) | 1 (25%) | 0 (0%) |
| Extremely | 0 (0%) | 0 (0%) | 0 (0%) | 0 (0%) | 0 (0%) | 0 (0%) |
| N/A | 21 | 17 | 22 | 15 | 15 | 17 |

| NEQ Item | Week 4 | | Week 8 | | Week 12 | |
|---|---|---|---|---|---|---|
| | **App** N = 26* | **GCBT** N = 19* | **App** N = 26* | **GCBT** N = 16* | **App** N = 19* | **GCBT** N = 18* |
| Item 17 | | | | | | |
| I was not affected | 1 (13%) | 0 (0%) | 1 (14%) | 0 (0%) | 0 (0%) | 0 (0%) |
| Slightly | 5 (63%) | 1 (50%) | 0 (0%) | 0 (0%) | 2 (33%) | 0 (0%) |
| Moderately | 2 (25%) | 1 (50%) | 5 (71%) | 1 (50%) | 2 (33%) | 2 (100%) |
| Very | 0 (0%) | 0 (0%) | 0 (0%) | 1 (50%) | 2 (33%) | 0 (0%) |
| Extremely | 0 (0%) | 0 (0%) | 1 (14%) | 0 (0%) | 0 (0%) | 0 (0%) |
| N/A | 18 | 17 | 19 | 14 | 13 | 16 |
| Item 18 | | | | | | |
| I was not affected | 2 (29%) | 2 (67%) | 1 (14%) | 0 (0%) | 1 (14%) | 1 (33%) |
| Slightly | 3 (43%) | 0 (0%) | 4 (57%) | 0 (0%) | 1 (14%) | 1 (33%) |
| Moderately | 0 (0%) | 1 (33%) | 0 (0%) | 0 (0%) | 3 (43%) | 1 (33%) |
| Very | 1 (14%) | 0 (0%) | 0 (0%) | 1 (100%) | 2 (29%) | 0 (0%) |
| Extremely | 1 (14%) | 0 (0%) | 2 (29%) | 0 (0%) | 0 (0%) | 0 (0%) |
| N/A | 19 | 16 | 19 | 15 | 12 | 15 |
| Item 19 | | | | | | |
| I was not affected | 0 (0%) | 0 (0%) | 1 (33%) | 1 (50%) | 1 (20%) | 0 (0%) |
| Slightly | 1 (33%) | 0 (0%) | 0 (0%) | 0 (0%) | 0 (0%) | 2 (50%) |
| Moderately | 1 (33%) | 1 (50%) | 1 (33%) | 0 (0%) | 3 (60%) | 2 (50%) |
| Very | 0 (0%) | 1 (50%) | 1 (33%) | 1 (50%) | 1 (20%) | 0 (0%) |
| Extremely | 1 (33%) | 0 (0%) | 0 (0%) | 0 (0%) | 0 (0%) | 0 (0%) |
| N/A | 23 | 17 | 23 | 14 | 14 | 14 |
| Item 20 | | | | | | |
| I was not affected | 1 (10%) | 0 (0%) | 2 (25%) | 1 (50%) | 1 (13%) | 1 (33%) |
| Slightly | 3 (30%) | 1 (50%) | 4 (50%) | 0 (0%) | 2 (25%) | 1 (33%) |
| Moderately | 4 (40%) | 1 (50%) | 0 (0%) | 0 (0%) | 3 (38%) | 1 (33%) |
| Very | 1 (10%) | 0 (0%) | 2 (25%) | 1 (50%) | 2 (25%) | 0 (0%) |
| Extremely | 1 (10%) | 0 (0%) | 0 (0%) | 0 (0%) | 0 (0%) | 0 (0%) |
| N/A | 16 | 17 | 18 | 14 | 11 | 15 |

* n (%). N/A: Not applicable or not available data.
